# Nutritional and Sensory Evaluation of Injera Prepared from tef and *Eragrostis curvula* (Schrad.) Nees. Flours with Sorghum Blends

**DOI:** 10.3389/fpls.2016.01059

**Published:** 2016-07-20

**Authors:** Habteab M. Ghebrehiwot, Hussein A. Shimelis, Kevin P. Kirkman, Mark D. Laing, Tafadzwanashe Mabhaudhi

**Affiliations:** ^1^African Centre for Crop Improvement, University of KwaZulu-NatalPietermaritzburg, South Africa; ^2^Department of Grassland Science, University of KwaZulu-NatalPietermaritzburg, South Africa; ^3^Department of Plant Pathology, University of KwaZulu-NatalPietermaritzburg, South Africa; ^4^Department of Crop Science, University of KwaZulu-NatalPietermaritzburg, South Africa

**Keywords:** *Eragrostis curvula*, injera, sensory evaluation, tef, underutilized crops

## Abstract

Injera is a fermented, sour bread consumed as a staple food in Eritrea and Ethiopia. The bread can be prepared from various cereals but tef [*Eragrostis tef* (Zucc.) Trotter] is the most preferred ingredient. This study assessed the acceptability of injera prepared using grains of a closely related but underutilized grass, *Eragrostis curvula* (Schrad.) Nees. The nutritive value of the grains was compared and the sensory attributes of injera made from flours of tef (control) and *E. curvula*, each combined with 0, 5, and 10% of sorghum flour, were assessed using a tasting panel. Nutrient analysis showed that *E. curvula* contains more than double the amount of crude protein found in tef. *E. curvula* also contains higher fat, dietary fiber and mineral nutrients than tef. Injera made of *E. tef* and *E. curvula* flours showed non-significant differences in taste, texture, appearance and overall acceptability. This suggest that *E. curvula* has the potential to serve as a novel source of gluten-free flour for human consumption. Agronomically viewed, growing *E. curvula* could be more advantageous for smallholder farmers on marginal lands because the species is a perennial that can produce a seed harvest twice a year, unlike tef, which is annual crop. It also tolerates acidic soils better than tef.

## Introduction

The challenges facing global food security due to increasing population, increasing pressure on finite land, and water resources and climate change calls for new and innovative solutions (Mabhaudhi et al., 2016a). This has led to suggestion that neglected and underutilized crops could be developed as alternatives to the current staple crops (Hammer and Heller, 1998; Mayes et al., 2012; Mabhaudhi et al., 2016b), especially under the arid and semi-arid conditions of sub-Saharan Africa (SSA; Chivenge et al., 2015). The emerging impetus to promote underutilized crops is mostly associated with their being an integral sub-set of agrobiodiversity, suitability to marginal production environments (Mabhaudhi et al., 2016a,b), often with a high nutritional value (Mabhaudhi et al., 2016a), attributes that can be used to promote food and nutrition security in marginal production areas.

In the course of human history, an estimated 7000 plant species have been cultivated for consumption at some point (FAO, 1998). However, humanity now relies primarily on maize, wheat, rice and soybean for protein and energy needs. Restoring diversity to cropping systems will be essential to achieving global food security (Hammer and Heller, 1998; Mayes et al., 2012; Mabhaudhi et al., 2016a) and building resilience to climate change (Padulosi et al., 2011; Chivenge et al., 2015; Mabhaudhi et al., 2016b). It will also contribute to increased levels of genetic diversity within cropping systems by breeding crops that can be cultivated under unfavorable conditions, such as drought, salinity, flooding, poor soils and extreme temperatures (Delgado et al., 2011; Mayes et al., 2012). However, despite reports of such potential, underutilized crops still remain under-researched, and the major crops continue to dominate agricultural landscapes.

*Eragrostis* is one of the largest and most widely distributed grass genera, with more than 350 species, adapted to a wide range of habitats (van den Borre and Watson, 1994). Although tef [*Eragrostis tef* (Zucc.) Trotter] is the only fully-domesticated species (Purseglove, 1976), many *Eragrostis* species have been harvested from the wild for millennia as valuable sources of grain (Brink and Belay, 2006). Oral history indicates that the seeds of some wild *Eragrostis* species such as *E. curvula, E. cilianensis, E. ciliaris, E. cylindriflora* Hochets*, E. gangetica* (Roxb.) Steud., *E. termula*, and *E. annulata* have been collected as a famine food in Africa (Duke, 1983; National Research Council, 1996; Brink and Belay, 2006). The seeds of *E. curvula* and *E. plana* have been used in making bread and beer (van Wyk and Gericke, 2000; Fish, 2003). Collectively, these accounts suggest that these underutilized wild *Eragrostis* species have the potential to contribute to the mix of food sources more than they currently do. Comparative studies on the morphological and cytological relationship of tef with other wild *Eragrostis* species suggest that these taxa could serve as a useful source of genes for the improvement of tef (Jones et al., 1978). Biochemical assessment of the relationship of tef and the wild *Eragrostis* species also showed many similarities (Bekele and Lester, 1981).

While tef may be of major importance in Ethiopia and Eritrea where it is the major source of flour used for preparing injera, it remains underutilized outside of these countries. There is a dearth of information on the agronomy, eco-physiology and nutritional value of these wild *Eragrostis* species. Currently, it is unknown whether wild *Eragrostis* species would offer adequate supplies of quality protein, mineral, fat and energy to local communities collecting the seeds of these species. If these underutilized *Eragrostis* species have any nutritional or agronomic advantages over conventional crops, then they could be used to diversify the global food basket, which would increase resilience in the global food system (Hammer and Heller, 1998; Mayes et al., 2012; Mabhaudhi et al., 2016a). Moreover, and in-depth knowledge of this wild species of the genus could provide an untapped reservoir of genetic diversity that could be used to improve tef.

The objective of this study was therefore to: (i) assess the nutritive value of *E. curvula* (seed/flour) in comparison to that of tef; (ii) assess the possibility of using *E. curvula* flour for the production of an acceptable quality injera; and (iii) to assess the overall acceptability of the new injera product through analysis of its sensory properties.

## Materials and methods

### Plant materials

Seeds (10 kg of grains) of *E. tef* (cultivar SA-Brown) and *E. curvula* (cultivar Ermelo) were purchased from McDonalds Seeds (Pty) Ltd, Pietermaritzburg, South Africa. The grains were independently stone-milled to a fine powder using Junior Mills (Pty) Ltd in Bloemfontein, South Africa. The flour was sieved to pass through a 0.05 mm mesh sieve and stored in an air tight container until used. Part of the flour was used for analyzing the chemical composition of the grains. For purposes of comparison, a sorghum-based flour Mabele Meal) was purchased from a local market in Pietermaritzburg, South Africa. Combining sorghum flour with tef flour has been shown to improve the sensory attributes of injera (Egli et al., 2004; Yetneberk et al., 2005).

### Preparation of the blends

*Eragrostis tef* and *E. curvula* flours were separately mixed with various proportions of sorghum flour (0, 5, and 10%; Table 1). The six blends were replicated three times each, yielding a total of 18 blended grain flour samples for injera baking. The blends were labeled with alphabets for identification and were kept in dry shelves at 25°C in the laboratory until used.

**Table 1:** **Composition of the six blends of flour prepared for making injera from tef flour and ***E. curvula*** flour combined with sorghum flour**.

**Samples**	***E. tef***			**Samples**	***E. curvula***		
	***E. tef* flour (g)**	**Sorghum flour (g)**	**Sorghum addition rate (%)**		***E. curvula* flour (g)**	**Sorghum flour (g)**	**Sorghum addition rate (%)**
A1	250	0	0	B1	250	0	0
A2	250	12.5	5	B2	250	12.5	5
A3	250	25	10	B3	250	25	10

### Dough making, fermentation, and injera preparation

Injera is made by mixing a cereal (e.g., tef, sorghum, barely, and blends thereof) flour with water to make a dough, and then triggering a fermentation process by inoculating the dough with *ersho*, a starter culture, left over from a previous fermentation. The starter culture is typically added at a ratio of 1:1.6 (w/v; Yetneberk et al., 2004; Baye et al., 2013). The fermentation usually lasts 2–3 days (depending on weather conditions), after which the dough is thinned into a batter before baking on an open platter.

In this study, injera was prepared as described by Yetneberk et al. (2004). The 18 flour blends (Table 1) were placed separately in 2 L ice-cream containers and subsequently made into dough by soaking in 500–600 mL of tap-water depending on the total weight of the blends. The dough was kept at room temperature (25°C) for 96 h. Fermentation was initiated by adding and appropriate volume of *ersho* into each container holding the blended flour. At the end of the fermentation process, the pH of the dough was measured using a glass electrode attached to a Horiba B-712 pH meter (Horiba Ltd, Kyoto Japan). Subsequently, the liquid layer that typically forms over the dough was gently poured off, leaving a semisolid dough.

After fermentation, 10% of the fermented dough was thinned with 100 mL of water and cooked in 200 mL of boiling water for 1 min. The gelatinized batter was cooled to ≈45°C at room temperature and added back to the fermenting dough. About 200 g of the fermented batter was poured in a circular manner onto a 45-cm diameter hot clay griddle, covered, and baked for approximate 2 min. The baked injera was then removed and kept in an airtight container.

### Sensory evaluation

**Figure 1:**
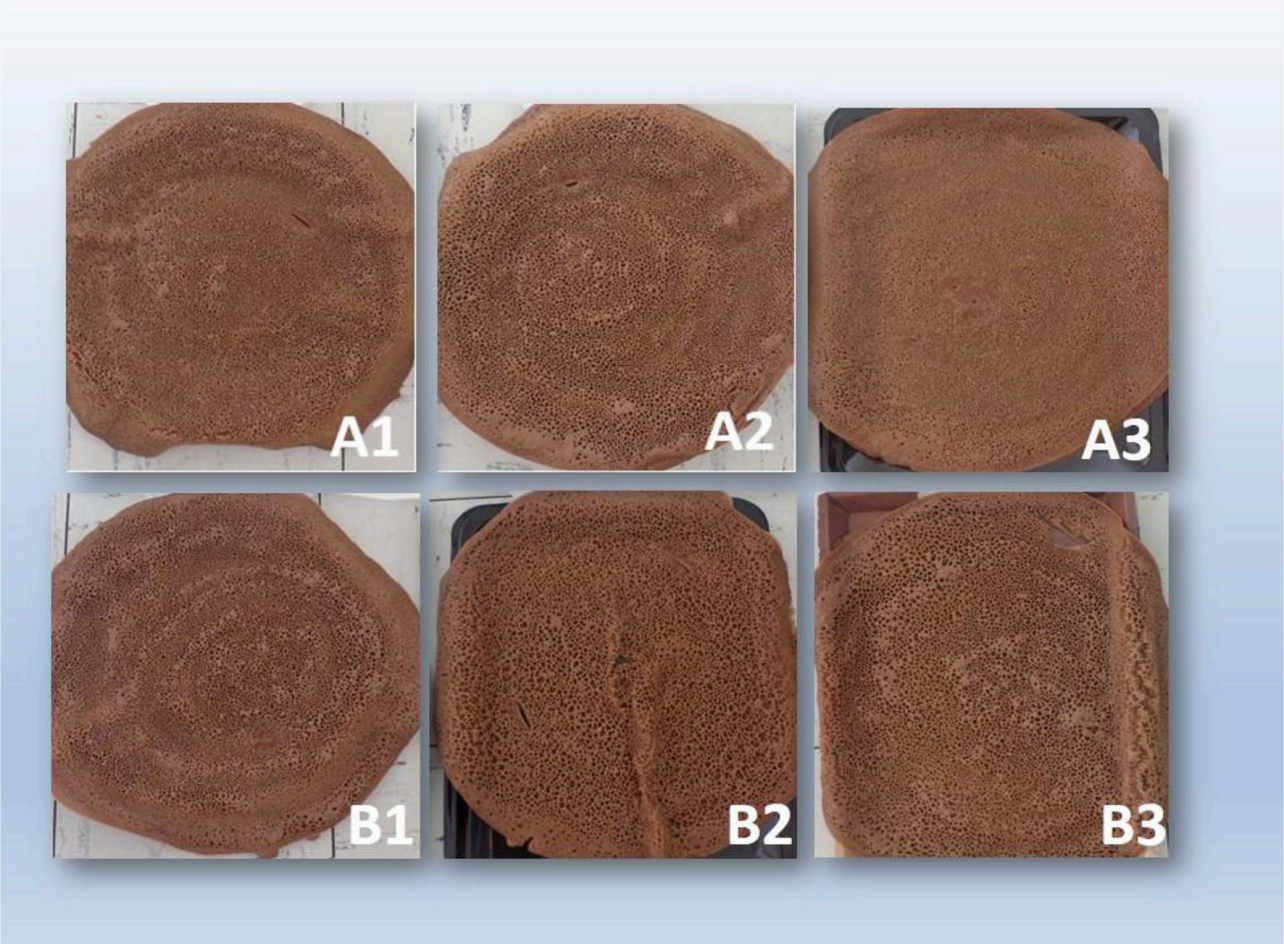
**The six injera products prepared from ***E. tef*** and ***E. curvula*** flours combined with 0, 5, and 10% of sorghum flour (Mabele Meal)**. **(A1)**, tef + 0% sorghum; **(A2)**, tef + 5% sorghum; **(A3)**, tef + 10% sorghum; **(B1)**, *E. curvula* + 0% sorghum; **(B2)**, *E. curvula* + 5% sorghum; **(B3)**, *E. curvula* + 10% sorghum.

In order to determine consumer acceptability of injera prepared using *E. tef* and *E. curvula*, a sensory evaluation was conducted. A semi-trained panel, consisting of 10 panelists (men and woman) who regularly consume injera as their staple food, was selected following the criterion described by Stone and Sidel (2004). It was believed that this panel can provide a technical judgment of acceptability useful to predict potential consumer preference. The panelists were provided with the randomly sequenced 18 samples (6 blends replicated 3 times each) for testing. They were asked to evaluate the products for taste, texture (mouth feel), appearance (eye size, honeycomb structure of the top surface of the injera) and overall acceptability. In this study color as a sensory parameter was excluded due to the close similarity of the products in color (Figure 1). All the samples were presented to panelists in a flat tray at ambient temperature (about 25°C) 2–4 h after baking. Since the panelists were not fully-trained, and to make the evaluation process consistent, a simple 5-point hedonic scale (questioner) was used, where 5 was extremely positive (like) and 1 extremely negative (dislike) for each sensory attribute. The panelists were provided with water to rinse their mouths after tasting each sample.

### Nutrient analysis

The determination of protein, fat and fiber was carried out using the Dumas method (dry combustion) on the Leco TruMac™ instrument (2010 LECO Corporation, Saint Joseph, Michigan, USA). This involved a total combustion of the matrix under oxygen. The gases produced were reduced by copper and then dried, while the CO_2_ was trapped. The nitrogen was then quantified using a universal detector. Mineral analysis was conducted using a Hunter apparatus (HCL extraction on the ICP), similar to that used for soil analysis.

### Statistical analysis

Data on the chemical composition of tef and *E. curvula* was subjected to a student *t*-test comparison using GenStat® (17th edition, VSN International, UK). The non-parametric data collected on taste, texture (mouth-feel), appearance and overall acceptability was analyzed using the Kruskal–Wallis H non-parametric test procedure. Means were compared using the non-parametric Mann–Whitney *U*-test procedure.

## Results and discussion

### Chemical composition of *E. tef* and *E. curvula* grains

The chemical composition of *E. tef* and *E. curvula* grains is presented in Table 2. Compared to *E. tef*, the seeds of *E. curvula* contained significantly (*P* < 0.001) higher levels of fat, ash, ADF (acid detergent fiber), NDF (natural detergent fiber) and more than double the amount of crude protein (18.47 ± 0.05 g/100 g sample). The measured values were higher than those measured in staple cereal crops such as rice, wheat, maize and sorghum (Moreno et al., 2014). The results of the current study were consistent with earlier reports (Jansen et al., 1962; Bekele and Lester, 1981). Bekele and Lester (1981) studied the variation in protein and amino acid composition both within and between 11 accessions tef varieties and 10 accessions of wild *Eragrostis* species, including *E. curvula*. They found that the wild species of *Eragrostis* had higher levels of protein and certain amino acids than the domesticated tef. This confirms that *E. curvula* is a nutritionally valid alternative to tef and other major cereal staples. The seeds of *E. curvula* also contained higher levels of minerals such as Zn, Cu, K, and Fe than *E. tef*. Higher levels of dietary fiber were also detectable in *E. curvula*, though it is unknown how such an amount of fiber would influence protein digestibility.

**Table 2:** **Nutrient analysis of whole grains of ***Eragrostis tef*** (cultivar SA-brown) and ***E. curvula*** (cultivar Ermelo) (On 100% dry basis)**.

**Composition**	**Unit**	***E. tef***	***E. curvula***	**Unpaired *t*-test and *p*-value**
Ash	g/100 g	2.49 ± 0.03	3.18 ± 0.03	*t*(*df* = 8) *t* = −16.9; *P* = 0.000^**^
Fat	g/100 g	2.64 ± 0.02	2.83 ± 0.05	*t*(*df* = 8) *t* = −3.48; *P* = 0.008^*^
ADF	g/100 g	7.50 ± 0.64	18.34 ± 0.10	*t*(*df* = 8) *t =* −16.8; *P* = 0.000^**^
NDF	g/100 g	11.78 ± 0.57	24.30 ± 0.29	*t*(*df* = 8) *t =* −19.5; *P* = 0.000^**^
Crude-protein	g/100 g	8.28 ± 0.04	18.47 ± 0.05	*t*(*df* = 8) *t =* −171; *P* = 0.000^**^
Ca	mg/100 g	0.19 ± 0.04	0.22 ± 0.00	*t*(*df* = 8) *t* = −0.842; *P* = 0.424^NS^
Mg	mg/100 g	354.18 ± 1.16	115.00 ± 0.63	*t*(*df* = 8) *t =* −26.0; *P* = 0.000 ^**^
K	mg/100 g	0.42 ± 0.06	0.584 ± 0.03	*t*(*df* = 8) *t* = −20.2; *P* = 0.000^**^
Na %	mg/100 g	0.01 ± 0.00	0.00 ± 0.00	*t*(*df* = 8) *t* = 1; *P* = 0.347^NS^
K/Ca+Mg	mg/100 g	0.44 ± 0.02	0.48 ± 0.00	*t*(*df* = 8) *t* = −2.12; *P* = 0.067^NS^
P	mg/100 g	0.42 ± 0.00	0.41 ± 0.05	*t*(*df* = 8) *t* = −0.11; *P* = 1.00^NS^
Zn	mg/100 g	37.30 ± 0.81	51.43 ± 0.07	*t*(*df* = 8) *t* = −17.0; *P =* 0.00^**^
Cu	mg/100 g	4.27 ± 0.08	10.02 ± 0.14	*t*(*df* = 8) *t =* −21.2; *P =* 0.00^**^
Mn	mg/100 g	354.18 ± 1.17	114.94 ± 0.64	*t*(*df* = 8) *t =* 193; *P =* 0.00^**^
Fe	mg/100 g	50.78 ± 1.08	84.53 ± 0.93	*t*(*df* = 8) *t =* −21.8; *P =* 0.00^**^

*Moisture % of samples as received by laboratory. NPN is non-protein nitrogen. ADFN and NDFN are calculated as nitrogen. Nitrogen % is calculated by dividing Protein by 6.25. The K/Ca + Mg should not be more than 2. Data on 100% dry matter basis*.

* *Significant difference at 5% level of significance*.

** *Highly significant difference at 1% level of significance*.

The flour of *E. tef* is gaining popularity in the Western World because of its attractive nutritional profile and its gluten-free nature. It can be used for the therapeutic treatment of patients with celiac disease (Moreno et al., 2014). However, grains of the pasture species, *E. curvula*, have superior levels of crude protein, dietary fiber, and minerals (Table 2). Therefore, cultivation and consumption of grains of *E. curvula* (also gluten-free) could be promoted in marginal production areas to contribute to food and nutrition security in these areas. In addition, its flour could also be promoted as an alternative to current gluten–free flour products.

### Sensory evaluation

Sensory evaluation is defined as the examination of a product (e.g., foods and beverages) through the evaluation of the attributes traceable by one or more of the five human senses—taste, smell, touch, sight, and hearing (Piana et al., 2004). It is used in food science to objectively analyse food quality. In many cases, it is an indispensable tool because it allows for the objective determination of whether or not consumers will accept a novel food product. Previous studies (Jansen et al., 1962; Bekele and Lester, 1981) that report on the nutritional value of *E. tef* and *E. curvula* did not evaluate its acceptability. This information is important for the successful promotion of *E. curvula* as a healthy alternative in the diets of people.

In the current study, a panel of 10 judges was used to describe the degree of consumer acceptance and satisfaction to the injera prepared using different combinations of *E. tef* and *E. curvula* flour, combined with sorghum flour. The taste of injera is associated with the sweet, sour and bitter sensations triggered in the mouth by contact with the injera. The sensory responses of the tasting panel to the injera prepared from six different blends of flours of sorghum, tef and *E. curvula* are provided in Table 3. Pair-wise comparisons of the products are given in Table 4. Out of the six injera samples, Sample A2 (tef + 5% sorghum) was the most preferred taste, scoring a 90% positive (like) response and the highest mean rating 4.6 followed by injera of Sample A1 (tef + 0% sorghum added) with a mean rating of 4.2. Pair-wise comparison between and among the injera prepared from tef (A1) and all the *E. curvula* flours (B1, B2, and B3) showed non-significant differences in taste. This implies that injera prepared using *E. curvula* flour tasted the same as the traditional injera prepared from tef flour.

**Table 3:** **Distribution of responses on a hedonic scale of 1–5 (bad to good), with resulting statistical indices for the six injera blends testing for taste, texture, appearance, and general acceptance**.

	**Assigned**	**Frequency of responses**					
	**value**	***E. tef***			***E. curvula***		
Injera varieties		A1	A2	A3	B1	B2	B3
pH		3.8	4.1	4.0	3.9	3.8	3.8
**FOR TASTE**							
Dislike very much	1	0	0	0	0	0	0
Dislike moderately	2	0	0	1	1	2	2
Neither like nor dislike	3	1	1	2	2	1	1
Like moderately	4	6	2	4	2	6	4
Like very much	5	3	7	3	5	1	3
Total responses		10	10	10	10	10	10
Mean rating		4.2^b^	4.6^a^	3.9^cd^	4.1^bc^	3.6^e^	3.8^de^
SE		1.14	1.30	0.71	0.84	1.05	0.71
% “Like” responses		90	90	70	70	70	70
**FOR TEXTURE**							
Dislike very much	1	0	0	0	0	0	0
Dislike moderately	2	0	0	0	0	1	0
Neither like nor dislike	3	1	0	1	1	0	3
Like moderately	4	4	1	6	4	8	5
Like very much	5	5	9	3	5	1	2
Total responses		10	10	10	10	10	10
Mean rating		4.4^b^	4.9^a^	4.2^b^	4.4^b^	3.9^c^	3.9^c^
SE							
% “Like” responses		90	100	90	90	90	70
**FOR APPEARANCE AND COLOR**							
Dislike very much	1	0	0	0	0	0	0
Dislike moderately	2	0	0	0	0	1	0
Neither like nor dislike	3	0	0	0	0	3	2
Like moderately	4	6	1	4	2	5	3
Like very much	5	4	9	6	8	1	5
Total responses		10	10	10	10	10	10
Mean rating		4.4^bc^	5.0^a^	4.8^ab^	4.8^ab^	3.8^d^	4.1^cd^
SE							
% “Like” responses		100	100	100	100	60	80
**FOR GENERAL ACCEPTANCE**							
Dislike very much	1	0	0	0	0	0	0
Dislike moderately	2	0	0	0	0	0	0
Neither like nor dislike	3	0	0	0	0	2	0
Like moderately	4	5	0	2	2	6	4
Like very much	5	5	10	8	8	2	6
Total responses		10	10	10	10	10	10
Mean rating		4.5^b^	5.0^a^	4.8^ab^	4.8^ab^	4.0^c^	4.6^ab^
SE							
% “Like” responses		100	100	100	100	80	100

*The injera were; tef (A1 = 0%, A2 = 5%, and A3 = 10% sorghum flour added) and Eragrostis curvula (B1 = 0%, B2 = 5%, and B3 = 10% sorghum flour added)*.

a–e *Mean rating values in the same row with shared letter(s) are not statistically different according to Duncan's multiple range test at 5% level of significance*.

**Table 4:** **Chi-square values comparing the six flour blends using the Kruskal–Wallis non-parametric test and Pair-wise comparison of the six injera using the Mann–Whitney ***U*** statistics for the four sensory parameters, Taste, Texture, Appearance, and Overall Acceptability**.

**No**.	**Pairs**	**Taste**	**Texture**	**Appearance**	**Overall acceptability**
1	A1 and A2	19.00^*^	25.00 ns	20.00^*^	25.00 ns
2	A1 and A3	49.5 ns	45.00 ns	30.00 ns	35.00 ns
3	A1 and B1	40.5 ns	34.00 ns	30.00 ns	35.00 ns
4	A1 and B2	37.00 ns	36.00 ns	31.00 ns	30.00 ns
5	A1 and B3	41.5 ns	36.50 ns	37.00 ns	45.00 ns
6	A2 and A3	27.00 ns	30.00 ns	40.00 ns	40.00 ns
7	A2 and B1	37.00 ns	40.00 ns	40.00 ns	40.00 ns
8	A2 and B2	12.00^*^	10.00^*^	10.00^*^	10.00^*^
9	A2 and B3	17.00^*^	10.00^*^	10.00^*^	30.00 ns
10	A3 and B1	43.00 ns	39.00 ns	50.00 ns	50.00 ns
11	A3 and B2	39.5 ns	31.50 ns	17.00^*^	18.00^*^
12	A3 and B3	43.00 ns	32.00 ns	19.00^*^	40.00 ns
13	B1 and B2	32.5 ns	19.00^*^	17.00^*^	18.00^*^
14	B1 and B3	36.00 ns	19.00^*^	19.00^*^	40.00 ns
15	B2 and B3	46.50 ns	49.5 ns	41.50 ns	26.00 ns
Chi-Square		11.63	18.46	24.93	19.45
Df		5	5	5	5
Significance level		0.040	0.020	0.000	0.002

* *Denotes significant difference and ns, non-significant difference both at 5% level of significance*.

The taste of Sample A3 (tef + 10% sorghum) was not significantly different from the taste of all the other injera prepared from *E. curvula* flour (B1, B2, and B3), and 70% of the panelist liked the taste of Samples A3, B1, B2, and B3. The dough of the six injera blends showed no significant difference in acidity. Combining tef flour with 5% sorghum flour significantly improved the taste and appearance of injera. By contrast, combining *E. curvula* flour with 5 and 10% sorghum flour caused a significant negative impact on the texture and appearance of injera (Table 4).

Texture is another important parameter often used to measure the quality of breads. It is determined by touch and refers to the degree of fluffiness, roughness, smoothness, hardness or softness. Out of the six samples, Sample A2 (tef + 5% sorghum added) was the most liked for texture, scoring a 100% positive (like) response, with the highest mean rating of 4.9, followed by Samples A1 (tef + 0% sorghum), A3 (tef + 10% sorghum), and B1 (*E. curvula* + 0% sorghum). 90% of the panelists liked the textures of injera of Samples A1, A3, B1, and B2. The lowest positive response was the injera of Sample B3 (*E. curvula* + 10% sorghum). Compared to the positive Control (Sample A1, tef flour with no sorghum added), the injera prepared from *E. curvula* flour with no sorghum added (B1) showed no significant difference in texture (Table 3) and were as likable as the Control. This indicates that the flour of *E. curvula* can be used to produce a well-textured injera. However, adding sorghum flour significantly decreased the quality of the texture of the injera made from *E. curvula* flour.

The appearance of injera is one of the most important parameters, which refers to the quality of the eyes (cells) of the honeycomb-like structure of the top surface of injera formed during cooking due to escaping CO_2_ bubbles (Yetneberk et al., 2005). The color of injera also affects the appearance of the injera in relation to its aesthetic appeal. In areas where injera is consumed as a staple food, (Eritrea and Ethiopia), people prefer their injera be white in color (Gebrekidan and GebreHiwot, 1982). In this study, all the injera prepared from *E. curvula* and tef (cultivar SA-brown) flours were brown in color (Figure 1). The sensory test showed significant differences (*P* < 0.001) in the appearances of the samples. The injera of Samples A2, A3, and B1 were the most preferred samples, followed by A1 and B6. The most interesting result was that injera prepared from 100% of *E. curvula* flour (Sample B1) was highly rated for its appearances similar to a classic injera. Even when blended with 5 and 10% sorghum flour, the appearance of injera prepared from *E. curvula* flour was liked by 60 and 80% of the panelists, respectively (Figure 1; Table 4).

Overall acceptability refers to the combinations of evaluations by consumers or panelists of a product. In this experiment, results showed that there was a statistically significant difference (*P* < 0.001) in the overall acceptability of the six injera samples. Injera of Samples A2, A3, B1, and B3 were the most acceptable followed by that of Sample A1. Similarly, 100% of the panelists liked all the injera prepared from tef (A1, A2, and A3). One hundred percentage of the panelists also accepted the overall qualities of injera prepared from two *E. curvula* flours (B1 and B3). However, Sample B2 (*E. curvula* + 5% sorghum) produced the least acceptable product, scoring a mean rating of 4.0 and an 80% overall acceptability (Table 4).

**Figure 2:**
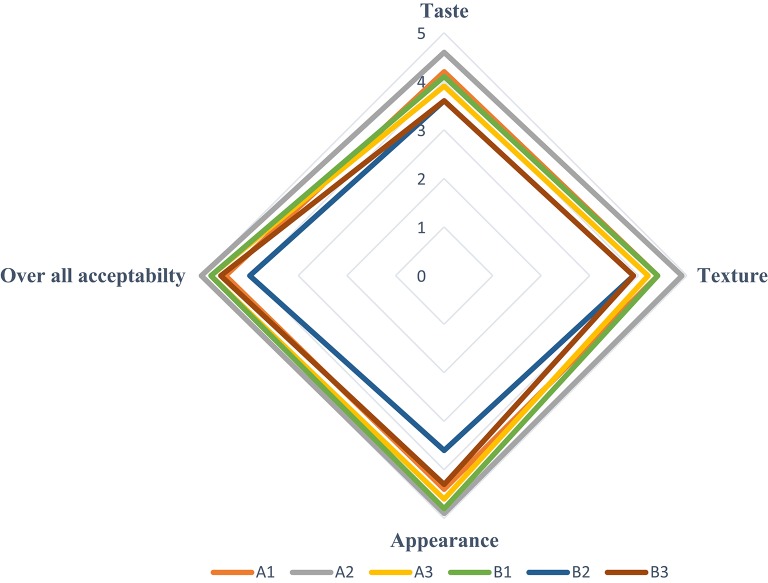
**Web-chart of the four sensory attributes of injera prepared from tef flour and ***E***. ***curvula*** flour blended with 0, 5, and 10% of sorghum flour**.

Taking all sensory attributes into account, though there was a statistically significant difference among samples, all blends scored a mean rating well above average (Table 3) which is an indicative of the goodness as products (Figure 2). The most preferred injera was produced from tef flour combined with 5% sorghum flour (Sample A2). However, the injera prepared from the flour of *E. curvula* also produced an excellent quality injera, especially when *E. curvula* flour with no sorghum was used (Sample B1). Apart from sorghum grains, grains of other crops may also be tried by blending in different proportion to prepare value added products from *E. curvula*.

## Conclusions

The present study revealed that grains of *E. curvula* contain high levels of protein, dietary fiber and minerals such as Fe and Mg, and that these values were substantially higher than tef and most other cereals. The injera (breads) made from flour of *E. curvula* had positive sensory attributes (taste, texture, appearance and overall acceptability) similar to those of the traditional injera made using tef flour. These findings suggest that beyond its current use as a pasture crop, flour from the grain of *E. curvula* could serve as an alternative source of food to produce high quality injera for human consumption, and could become a valued gluten-free flour globally. It is also possible that the grains of *E. curvula* could serve as a raw material for other food products such as porridge, biscuits, muffins, beer and beverages. Given that *E. curvula* is a drought resistant perennial grass compared to tef (annual grass), and given its hardiness and tolerance of acid soils, there is scope to promote its cultivation and utilization in semi-arid areas of sub-Saharan Africa where other major cereal staples do not perform well. Even in Ethiopia and Eritrea, it could be grown in regions with severe soil acidity that limits tef production. However, further studies are required to evaluate the nutritional qualities, health benefits and grain productivity of *E. curvula* as necessary steps toward developing it as a productive, nutritious grain crop that can be grown under semi-arid conditions.

## Author contributions

The idea of testing the grains of *Eragrostis curvula* for injera making was first conceived by KK. Afterwards, ML, SH, and HG designed and carried out a series of experiments. The manuscript was prepared by HG and TM. The manuscript was edited by all authors.

### Conflict of interest statement

The authors declare that the research was conducted in the absence of any commercial or financial relationships that could be construed as a potential conflict of interest.
